# Room-temperature efficient light detection by amorphous Ge quantum wells

**DOI:** 10.1186/1556-276X-8-128

**Published:** 2013-03-16

**Authors:** Salvatore Cosentino, Maria Miritello, Isodiana Crupi, Giuseppe Nicotra, Francesca Simone, Corrado Spinella, Antonio Terrasi, Salvatore Mirabella

**Affiliations:** 1 MATIS IMM-CNR and Dipartimento di Fisica e Astronomia, Università di Catania, via S. Sofia 64, Catania 95123, Italy; 2 IMM-CNR, VIII strada 5, Catania 95121, Italy

**Keywords:** Germanium, Nanostructures, Light absorption, Quantum confinement effect

## Abstract

In this work, ultrathin amorphous Ge films (2 to 30 nm in thickness) embedded in SiO_2_ layers were grown by magnetron sputtering and employed as proficient light sensitizer in photodetector devices. A noteworthy modification of the visible photon absorption is evidenced due to quantum confinement effects which cause both a blueshift (from 0.8 to 1.8 eV) in the bandgap and an enhancement (up to three times) in the optical oscillator strength of confined carriers. The reported quantum confinement effects have been exploited to enhance light detection by Ge quantum wells, as demonstrated by photodetectors with an internal quantum efficiency of 70%.

## Background

Due to its large compatibility with Si technology and to its pseudodirect bandgap, germanium has recently drawn a vast scientific concern for promising electronic and photonic applications [1-5]. In particular, quantum confinement [1,6] and tensile strain [2-4] effectively modify the electronic bandgap of crystalline (c-) Ge, in such a way that it opens the route for Si-compatible, room-temperature operable devices as optical modulators [1,2] or lasers in the commercial C-band [4]. Quantum confinement effects (QCE) appear in Ge nanostructures (NS) more conspicuously than those in Si due to the much larger exciton Bohr radius (approximately 24 nm in Ge compared with approximately 5 nm in Si) [7,8] which allows the tuning of the QCE to greater extents. Photoluminescence peak coming from excitons confined in Ge nanocrystals exceeds the bandgap of Ge bulk $\left(E_{G}^{\mathrm{bulk}}\right)$ by an energy amount much larger than that for Si nanocrystals [9]. Still, all these effects have been extensively proven for c-Ge NS, while the light interaction with amorphous (a-) NS of Ge was poorly investigated. Moreover, fabrication of amorphous materials is typically less expensive than that of crystalline materials due to lower synthesis temperatures, higher deposition rates, and cheaper substrates. Thus, the chance to exploit QCE in a-NS represents a key question for bandgap engineering in confined materials.

Amorphous Si quantum dots (QDs) [10] and quantum wells (QWs) [11,12] showed significant size dependence in bandgap (*E*_*G*_) tuning, well modeled within the effective mass theory by the following relation:

(1) $$E_{G}=E_{G}^{\mathrm{bulk}}+\frac{A}{L^{2}},$$

where *L* is the NS size and *A* = *π*^2^*ћ*^*2*^/2m* is the confinement parameter (m* is the electron-hole pair effective mass) [12]. Actually, the generally accepted picture of the electronic energy bands in a-Si is quite similar to that of c-Si, except for the presence of significant band tails and localized states within the gap, both originating from defects in the a-structure [13]. Even if electronic states are extended or localized (weakly or strongly) and the **k** vector conservation is thus released, the effective mass theory has still been successfully applied when effective masses are considered as parameters giving average effects in a nonregular lattice [12,13]. Within this scenario, the confinement parameter (*A*) found for a-Si QDs (2.40 eV·nm^2^[10]) is larger than that for a-Si QWs (0.72 eV·nm^2^[13]), as expected due to the larger 3D confinement [10,14]. As far as a-Ge NS are concerned, some size-dependent shift of *E*_*G*_ was evidenced in amorphous Ge/SiO_*x*_ superlattices deposited by vacuum evaporation [15]; however, no evaluation of the extent of quantum confinement has been reported, and no studies are present on their potential application for light harvesting purposes. This chance, added to the pseudodirect bandgap of Ge and to its higher absorption coefficient with respect to Si, makes a-Ge NS very attractive both for fundamental studies and for efficient visible light detection [16,17].

In this letter, we report on the large bandgap tuning observed at room temperature in amorphous Ge QWs (2 to 30 nm in thickness) due to quantum confinements effects. This process has been successfully modeled, evidencing a significant increase of the optical oscillator strength and a confinement parameter (*A* = 4.35 eV·nm^2^) much larger than that previously reported in a similar a-Si NS [10,13]. Finally, we have proven the use of a-Ge thin films as the active absorber in photodetectors, demonstrating the chance of using Ge QWs as efficient photosensitizer.

## Methods

**Figure 1:**
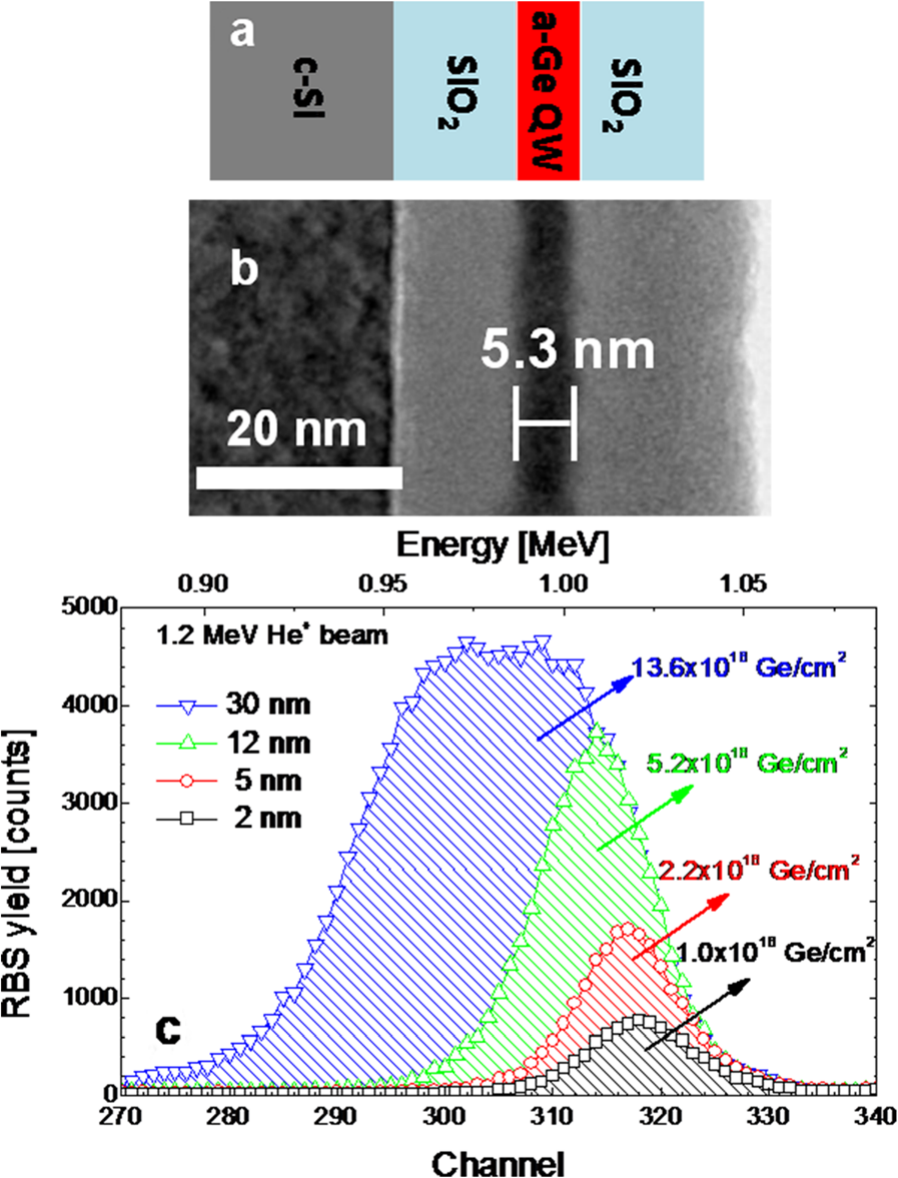
**Schematic, TEM images, and RBS spectra of samples.** (**a**) Schematic of sample structure, (**b**) cross-sectional bright-field Z-contrast TEM images of 5-nm-thick a-Ge QW sample, and (**c**) RBS spectra of a-Ge QWs. The filled areas are proportional to the Ge content of each QW (from 1.0×10^16^ Ge/cm^3^ to 13.6×10^16^ Ge/cm^3^) as reported in the figure.

On (001) *n*-doped Si wafer or on fused silica quartz, a SiO_2_/Ge/SiO_2_ structure has been deposited at room temperature by magnetron sputtering technique (pre-deposition base pressure of 1 × 10^−9^ mbar and argon pressure during deposition of 5 × 10^−3^ mbar), using high-purity Ge and SiO_2_ targets. The Ge deposition rate was fixed at 1 nm/min, and the thickness of the a-Ge QW was varied in the range of 2 to 30 nm. Top and bottom SiO_2_ films (approximately 10-nm-thick each) were used as barriers for the QW structure, as schematized in Figure 1a. Cross-sectional transmission electron microscopy (TEM), used to evaluate the roughness and thickness of the QWs, was performed with a JEOL 2010 F microscope (JEOL Ltd., Akishima, Tokyo, Japan) operating at 200 kV equipped with a Schottky field-emission gun and an ultrahigh-resolution objective lens pole piece. Rutherford backscattering spectrometry (RBS) was employed to measure the Ge dose contained in each sample and the stoichiometry of the barrier layers. A glancing detection mode was used (1.2 MeV He^+^ beam, 98° backscattering angle) to enhance the depth resolution. Light absorption spectroscopy was done on samples deposited onto the quartz substrate by measuring the transmittance (*T*) and reflectance (*R*) spectra in the 200- to 2,000-nm wavelength range with a Varian Cary 500 double-beam scanning UV/visible/NIR spectrophotometer (Varian Medical Systems, Palo Alto, CA, USA). With the same growth conditions, we deposited a control sample (SiO_2_ layer without Ge film) and verified by RBS and ellipsometry that it has the correct SiO_2_ stoichiometry and that it is truly transparent in the 200- to 2,000-nm range. The a-Ge QW samples were used to make basic photodetector devices to perform room-temperature photocurrent measurement. A metal-insulator-semiconductor (MIS) configuration was pursued after sputter deposition at room temperature of a transparent gate electrode (Al-doped ZnO, 3 mm in diameter) onto the SiO_2_/Ge/SiO_2_ structure grown upon *n*-Si substrate. Finally, silver paint was used to assure the electrical back contact. A 250-W tungsten halogen lamp, equipped with an optical monochromator and a 19-optical fiber bundle, provided white or wavelength-dispersed illumination on the sample in the 400- to 1,100-nm range with a photon flux in the range of 10^13^ to 10^14^ photons/(cm^2^·s), while a Keithley 4200 semiconductor characterization system (Keithley Instruments Inc., Cleveland, OH, USA) was used for the current-voltage curves.

## Results and discussion

The structural characterization of a-Ge QWs is summarized in Figure 1. If relevant fractures occurred in the Ge film, the quantum confinement would change from one-dimensional (1D) regime to two-dimensional (2D) or three-dimensional (3D) regimes, as the unconfined feature of the electron wave functions in the plane parallel to the surface would be lost. Such circumstances have been denied by extensive TEM and HRTEM investigation performed both in plan and in cross-sectional view. As an example, a TEM image is reported in Figure 1b for the 5-nm a-Ge QW sample (grown on Si substrate), showing SiO_2_ films (brighter layers) embedding the Ge QW (thin darker layer). The measured thickness, *d*, and roughness of the a-Ge QW are 5.36 and 3.65 nm, respectively. This means that even if some sparse thinning of the Ge QW occurs, the electronic wave functions are still confined only in the growth direction, preserving the 1D confinement regime. Similar considerations can be done for all the a-Ge QW samples. Figure 1c reports the RBS data in the 0.88- to 1.09-MeV energy range which is relative to He^+^ backscattered from Ge atoms. The peak area was converted into Ge atomic dose contained in each QW, as indicated in the figure. By combining these data with the thickness measured by TEM, we obtain a density of 4.35 × 10^22^ Ge atoms/cm^3^, which is in agreement with that of bulk Ge (4.42 × 10^22^ atoms/cm^3^) [18]. This last evidence clearly indicates the absence of low-density regions or voids in the as-deposited a-Ge films.

**Figure 2:**
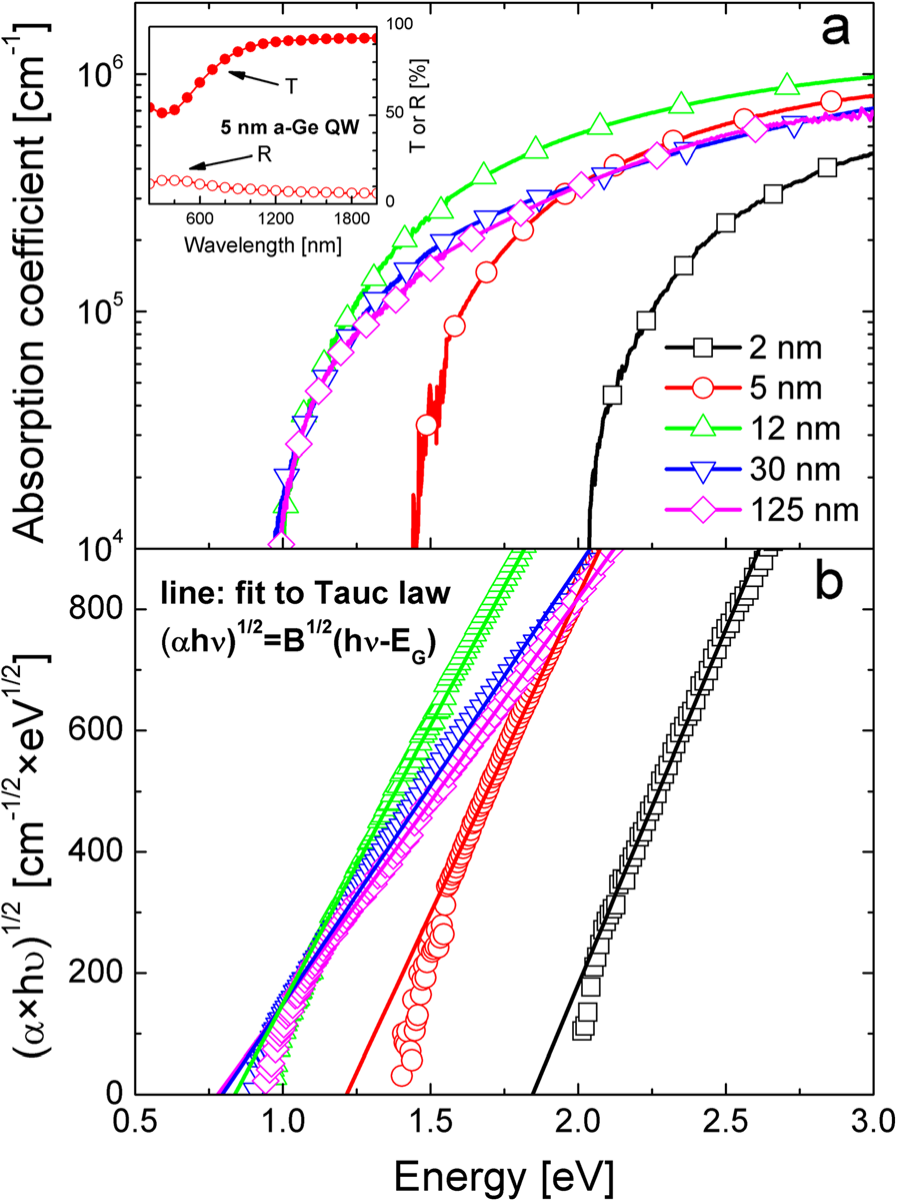
**Absorption coefficient spectra and Tauc plots and relative linear fits.** (**a**) Transmittance and reflectance spectra of 5-nm a-Ge QW (inset). Absorption coefficient of a-Ge QW of different thicknesses together with the spectrum of a bulk-like 125-nm a-Ge. (**b**) Tauc plots (symbols) and relative linear fits according to the reported Tauc law (lines).

To ascertain if quantum confinement affects the energy gap of a-Ge QWs, light absorption spectroscopy was performed in the samples grown on quartz substrates. Accurate *T* and *R* measurements (some of which are reported in the inset of Figure 2a) have been performed at room temperature to extract the absorption coefficient (*α*) of such thin Ge films, as described in another study [19]. The overall indetermination on *α*, also including errors on *d*, *T*, and *R*, is about 5%, while the dynamic range of the product *αd* was 1 × 10^−3^ to 2 × 10^−1^. Figure 2a shows the *α* spectra of the a-Ge QWs and of an a-Ge film (125-nm thickness) used as a reference in a bulk, unconfined film. The absorption coefficient of the 30-nm a-Ge QW is similar to that of the 125-nm a-Ge sample, both evidencing an absorption edge at about 0.8 eV, typical of an a-Ge bulk [20]. On the contrary, by decreasing the thickness of the a-Ge QW from 12 to 2 nm, an evident blueshift occurs in the onset of the absorption spectrum. Moreover, in the 12-nm a-Ge QW, the *α* spectrum is higher than in the 30-nm a-Ge QW sample, despite the similar onset. Therefore, under 30 nm, the thickness of the a-Ge QW clearly affects the photon absorption mechanism as an effect of spatial confinement on the electronic energy bands. Actually, the Bohr radius for excitons in Ge is about 25 nm [7,21], and thus, the observed variation in the absorption spectra can be thought as a quantum confinement effect on the energy band in a-Ge QWs. To deepen this point, a proper description of the light absorption mechanism in the a-NS is needed.

In bulk amorphous semiconductors, *α* at energy *hν* is proportional to $\frac{J_{c,v}\left(\mathrm{h\nu}\right)\times M^{2}}{\mathrm{h\nu}}\;$[22,23], where *J*_*c*,*v*_(*hv*) is the joint density of states separated in energy by *hν*, and *M* is the matrix element of optical transition, accounting for the overlap integral of electron-hole wave functions and nearly constant for visible photons [23]. Under the assumption of parabolic band edges for valence and conduction bands, one gets $J_{c,v}\left(\mathrm{h\nu}\right)\propto {\left(\mathrm{hv}-E_{G}^{\mathrm{bulk}}\right)}^{2}$[22]; thus, for α values larger than 1 × 10^4^ cm^−1^, the energy dependence of *α* is satisfactorily modeled by the Tauc law:

(2) $$\alpha =\frac{B}{\mathrm{h\nu}}\;{\left(\mathrm{h\nu}-E_{G}^{\mathrm{bulk}}\right)}^{2},$$

where the Tauc coefficient, *B*, includes *M*^*2*^[22,23]. In the a-NS, Equation 2 can be used if size effects are properly considered, such as bandgap widening (acting on *E*_*G*_) or enhanced oscillator strength (*O*_*S*_, which increases *M*^*2*^, and then *B*) [6]. If the Tauc law properly describes the light absorption, (*αhν*)^1/2^ versus *hν* (called Tauc plot) gives a linear trend in the energy range for which *α* > 1×10^4^ cm^−1^, as it clearly occurs for all the a-Ge QWs (Figure 2b). The application of Tauc law to a-Ge QWs allows to determine *B* and *E*_*G*_ through linear fitting procedures (lines in Figure 2b). By reducing the QW thickness down to 2 nm, *E*_*G*_ (fit intercept with energy axis) shifts at higher energy and *B* (square of the fit slope) increases. These findings confirm the quantum confinement effect in a-Ge QWs. In fact, no variations of the electronic band diagram are expected above the Bohr radius, while below it, a broadening of energy levels shifts *E*_*G*_ to larger values. In addition, the stronger spatial confinement of carriers in very thin a-Ge films leads to excitonic absorption enhancement, which is observed as the increase of *B*. This evidence clearly points out that light absorption can be profitably enhanced by the quantum confinement in a-Ge QWs, confirming the previous indication of another study [15]. In order to quantify the bandgap widening and the excitonic effects, further analyses have been done.

**Figure 3:**
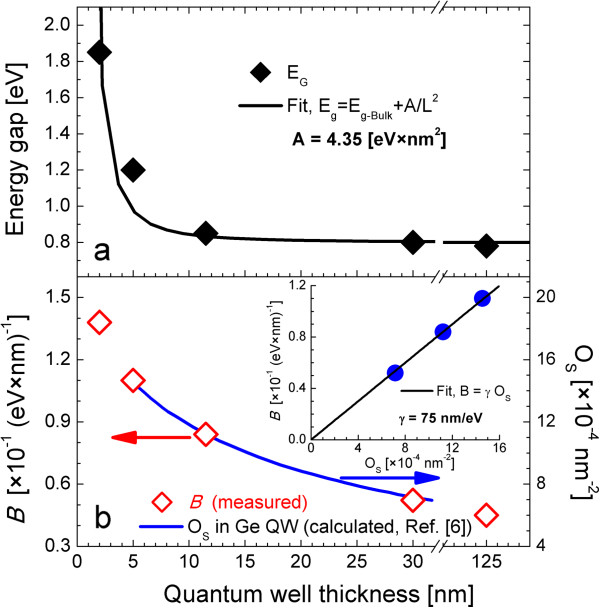
**Experimental and theoretical values of energy gap and *B*.** (**a**) Experimental values (diamonds) of energy gap in a-Ge QW versus thickness, fitted through effective mass theory (solid line). (**b**) Experimental values of *B* (diamonds, left axis) compared with the calculated trend [9] for the oscillator strength (*O*_*S*_) in Ge QWs (line, right axis). Inset shows the linear correlation between *B* and *O*_*S*_.

Figure 3 describes the quantum confinement effects in the light absorption process in a-Ge QWs. Figure 3a demonstrates the dependence on the QW thickness of the optical bandgap (diamonds) evidencing a blueshift up to 1 eV for the 2-nm sample. Previous data on amorphous Ge/SiO_*x*_ superlattices reported much lower blueshifts of *E*_*G*_ (only about 0.1 eV for the same thickness) most likely due to the use of nonstoichiometric SiO_*x*_ as barrier, giving a weaker confinement effect in comparison to SiO_2_[15]. Our *E*_*G*_ data have been fitted (solid line) within the effective mass theory assuming an infinite barrier by Equation 1, with *A* being the only fit parameter. $E_{G}^{\mathrm{bulk}}$ was fixed as the bandgap of bulk a-Ge (0.8 eV, [20]), which is also in good agreement with our value for 30-nm QWs. The good fit agreement with experimental data confirms that the shift in the energy gap is ascribed to QCE and that SiO_2_ layers act as infinite potential barrier, ensuring a strong confinement of electrons within Ge QWs. Moreover, the experimental confinement parameter in a-Ge QWs resulted to be 4.35 eV·nm^2^, which is not so far from the theoretical value of 1.97 eV·nm^2^ reported by Barbagiovanni et al. for a strong quantum confinement in c-Ge QW [14]. Our value of *A* for a-Ge QWs is also much larger than that measured in a-Si QWs (0.72 eV·nm^2^[12]), evidencing the bigger effect of quantum confinement in Ge NS. Actually, *A* is given by *A* = *π*^2^*ћ*^*2*^/2m*, where m* is the reduced effective mass of excitons, expected to be approximately 0.1 × *m*_e_ in Ge (*m*_e_ is the electron mass), which is five times smaller than that in Si (0.48 *m*_e_) [7,14,24]. In the a-Si NS, the *A* parameter was observed to increase by a factor of 3 going from 1D (QWs) to 3D (QDs) structures ([10,12]); thus, in a-Ge QDs, the confinement parameter is expected to overcome the huge value of 13 eV·nm^2^.

Figure 3b reports on the increase in the light absorption efficiency due to confinement. In fact, beyond the energy blueshift, another interesting effect of the spatial confinement is the enhanced interaction of light with confined carriers. On the left axis of Figure 3b, the variation of *B* with QW thickness is plotted, as extracted from fits in Figure 2b. Such a quantity significantly increases up to three times going from bulk to the thinnest QW, evidencing the noteworthy increase of the light absorption efficiency. In fact, the thinner the QW thickness, the smaller is the exciton Bohr radius, thus giving rise to a larger oscillator strength (*O*_*S*_) [6]. Such an effect was predicted and observed for c-Ge QWs [6], but now, for the first time, it is experimentally assessed also in a-Ge QWs. Since the *B* parameter in Equation 2 includes the matrix element of optical transition *M* (which is related to *O*_*S*_), the increase in *B* can be thought as the evidence of the enhanced oscillator strength in the confined system. Indeed, in Figure 3b, on the right axis, the variation of *O*_*S*_ with thickness in the c-Ge QW is reported, as calculated in the 5- to 35-nm thickness range by Kuo and Li, using a 2D exciton model and infinite barrier [6]. The good agreement between measured *B* and calculated *O*_*S*_ is the experimental confirmation that the enhanced absorption efficiency observed at room temperature in a-Ge QWs is actually due to the excitonic effect. The inset of Figure 3b evidences the linear correlation between *B* (measured at 5, 12, and 30 nm) and the expected *O*_*S*_ (for those thicknesses), allowing for the estimation of the factor of proportionality (*γ* = *B*/*O*_*S*_, which accounts for the absorption efficiency normalized to the oscillator strength). Thus, a proper modeling applied to light absorption measurements at room temperature allowed to quantify the extent of size effect in a-Ge QWs and to disentangle the oscillator strength increase and the bandgap widening in these structures.

**Figure 4:**
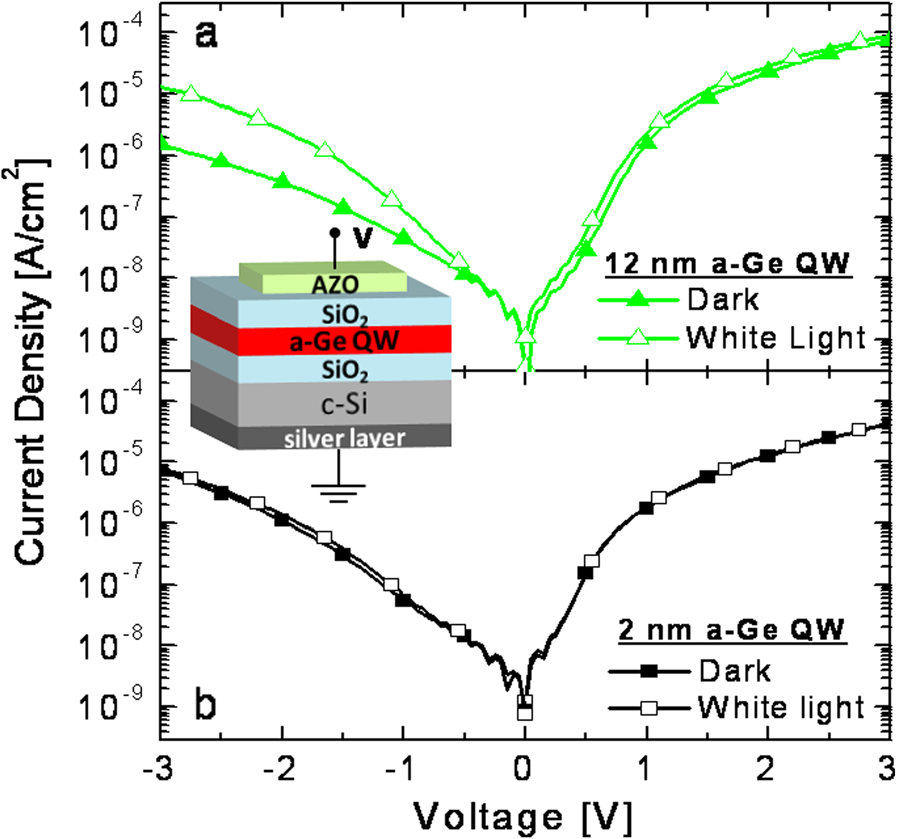
**Current density versus voltage measurements.** They are under dark (filled symbols) or white light (empty symbols) conditions, in devices containing (**a**) 12- or (**b**) 2-nm a-Ge QWs. The used metal-insulator-semiconductor configuration is drawn in the figure.

In order to test if photogenerated carriers in a-Ge QWs can be separated and collected through the action of an external electric field, we realized a photodetector device, as illustrated in the drawing of Figure 4, and performed transversal current density versus voltage (*J*-*V*) measurements in dark and under white light illumination conditions. Figure 4 reports the *J*-*V* curves for samples with 12-nm (Figure 4a) or 2-nm (Figure 4b) a-Ge QW. In dark conditions, both the MIS devices (biased as shown in the drawing) have similar behavior in forward and reverse biases. Most of the applied voltage is dropped across the dielectric (SiO_2_) stacks, while the QW thickness slightly lowers the dark current density (*J*_dark_) in the thicker sample (offering a more resistive path). Upon white light illumination, *J*-*V* values remain largely unaffected in the forward bias, while an increase of the current density (*J*_light_) occurs for the thicker samples in the reverse bias regime. In particular, for a negative bias of −3 V, the net photocurrent (*J*_light_ − *J*_dark_) increases from 1 to 12 μA/cm^2^ going from 2 to 12 nm of QW thickness. The net photocurrent is due to the electron-hole pairs photogenerated in the QW and in the substrate (*n*-Si). As the device is reverse biased, electrons are pushed to the substrate and holes to the transparent electrode. It should be noted that by increasing the Ge QW thickness, the contribution of the substrate to the net photocurrent shrinks. In fact, the photogeneration of electron-hole pairs in the substrate decreases because of the light absorbed in the QW, and the carrier collection lowers because of the higher resistance. By comparing the images in Figure 4a,b, we can appreciate the role of the a-Ge film, as the MIS devices differ only for the QW thickness. The higher net photocurrent measured in the thicker QW gives a clear evidence of a positive photoconductivity effect within a-Ge QWs.

**Figure 5:**
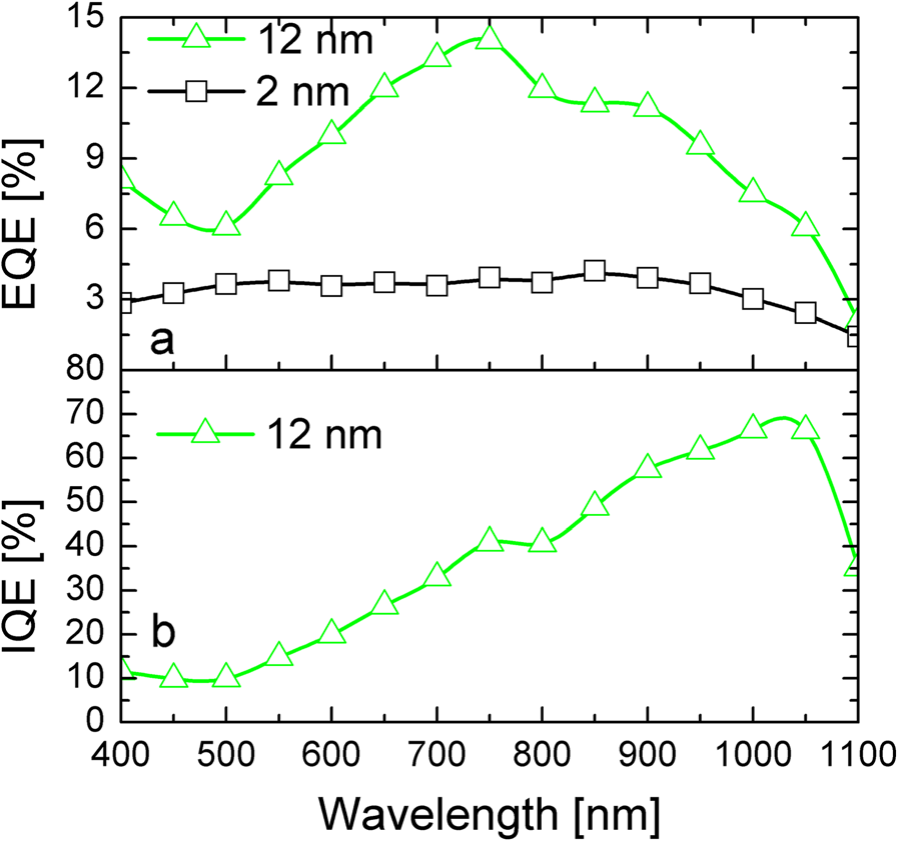
**EQE and IQE spectra.** (**a**) EQE spectra taken at −3-V bias for the 2- or 12-nm a-Ge QW devices. (**b**) IQE spectrum for the 12-nm a-Ge QW photodetector biased at −3 V.

In order to quantitatively investigate the spectral response of the devices, we illuminated them with different wavelengths and measured the external quantum efficiency ($\mathrm{EQE}=\frac{\mathrm{hc}}{\lambda }\left(J_{\mathrm{light}}-J_{\mathrm{dark}}\right)/P,$ where *P* is the power of incident photons per unit area), which gives the number of collected carriers per incident photon at a given wavelength. In Figure 5a, the EQE spectra are reported for both the devices biased at −3 V. The device with 2-nm a-Ge shows a fairly low and flat photoresponse in all the investigated spectral range. Such a response was expected after the very low net photocurrent reported in Figure 4b. Actually, this behavior can be mainly attributed to the contribution of the carrier generation and extraction within the depleted region layer in the Si substrate, without a significant role of the Ge QW since (1) light absorption by the 2-nm a-Ge QW occurs only for photons with energy larger than 1.8 eV (*λ* ≤ 700 nm) and (2) even for *λ* ≤ 700 nm, the fraction of absorbed light is only a few percent of the total incident light (Figure 2a). Thus, a really small contribution of the 2-nm a-Ge QW is expected on the overall response of the photodetector, allowing for the consideration of the 2-nm a-Ge QW device as a reference for the substrate behavior. On the contrary, the device with 12-nm a-Ge QWs shows a much larger EQE, clearly indicating the paramount role of carrier photogeneration within a-Ge films. Even if the maximum EQE is only 14%, one should consider that the photoresponse in this device is mainly attributable to the photocarrier generation within the 12-nm Ge layer and their following extraction, since the Si substrate has only a minor contribution in this case. In particular, the fraction of absorbed light in the 12-nm-thick a-Ge QW is much lower than unity in the entire spectral range investigated, since we have already reported the absorption spectrum of this same sample (Figure 2a). Therefore, we can extract the internal quantum efficiency (IQE), which gives the number of collected carriers per absorbed photon at a given wavelength by the Ge layer, $\mathrm{IQE}=\frac{\mathrm{hc}}{\lambda }\left(J_{\mathrm{light}}-J_{\mathrm{dark}}\right)/\left[P\left(1-e^{-\alpha \cdot d}\right)\right]$. As reported in Figure 5b, the IQE shows values as high as 70% in the near-infrared region, close to the *E*_*G*_ (approximately 0.9 eV) that we measured for this sample through an independent method in Figure 2b. This correlation further supports the main role of the a-Ge QW as active absorbing layer in the photodetector device. The IQE spectrum decreases for higher photon energy as the collection of the hotter carriers is less probable due to recombination issues.

The high IQE value indicates that almost every absorbed photon can be converted in an electrical signal and detected in this simple photodetector device. Hence, the high IQE measured on this sample reveals that a-Ge QWs can be profitably used as efficient photosensitizer in light detection devices. In fact, the excitonic effect and the bandgap tuning due to the quantum confinement effect can be further exploited to realize tunable and efficient photodetectors operable at room temperature, which are compatible with Si technology and with low-cost approach.

## Conclusions

In this work, we reported on the large quantum confinement effects shown by single amorphous Ge ultrathin (2- to 30-nm thicknesses) films embedded in SiO_2_ barrier layers. These confined structures, grown by magnetron sputtering at room temperature, revealed a large blueshift (about 1 eV) in the optical bandgap and a significant increase (up to three times) in the light absorption efficiency due to an enhanced optical oscillator strength. Such effects, typically observed at cryogenic temperature or in crystalline materials, are now evidenced in the amorphous phase and at room temperature for Ge and have been fully explained by the Tauc model joined with the effective mass theory. Moreover, these a-Ge quantum wells have been employed as proficient light sensitizer in a basic photodetector device, showing at room temperature an enhanced photocurrent, with an internal quantum efficiency as high as 70%. This datum and the noteworthy excitonic effect, evidenced here, open the route for application of a-Ge QWs in efficient and low-cost light detectors.

## Competing interests

The authors declare that they have no competing interests.

## Authors’ contributions

SC contributed to sample processing, characterization, and data analysis and interpretation and drafted the manuscript. SM conceived the study, contributed to sample characterization, and data analysis and interpretation and revised the manuscript. IC contributed to the electrical characterization and data interpretation. MM synthesized the samples. GN and CS provided TEM analysis. FS contributed to optical analysis. AT conceived the study, contributed to data interpretation, and coordinated the work. All authors read and approved the final manuscript.
